# A Systematic Review of Independent and Chain Pharmacies Effects on Medication Adherence

**DOI:** 10.3390/pharmacy10050124

**Published:** 2022-09-29

**Authors:** James Nind, Alesha Smith, Shane Scahill, Carlo A. Marra

**Affiliations:** 1 School of Pharmacy, University of Otago, Dunedin 9016, New Zealand; 2 School of Pharmacy, University of Auckland, Auckland 1010, New Zealand

**Keywords:** community pharmacy, adherence, independent pharmacy, chain pharmacy, access to medicine, medicine use

## Abstract

As the last step in the care pathway, pharmacies can significantly impact a patient’s medication adherence and the success of treatment. The potential impact of patient’s pharmacy choice on their medication adherence has yet to be established. This study aims to review the impact a pharmacies ownership model, either independent or chain, has on its users’ medication adherence. As a generalisation, independent pharmacies offer a more personal service and chain pharmacies offer medications at lower prices. A keyword search of EMBASE and MEDLINE databases in March 2022 identified 410 studies, of which 5 were deemed to meet our inclusion criteria. The studies mostly took place in North America, measured medication adherence using pharmacy records over a 12-month period. This review was unable to substantiate a difference in the rate of medication adherence between the users of independent and chain pharmacies. However, those with a lower income, greater medication burden, and increased age appeared to use an independent pharmacy more than a chain pharmacy and to have greater medication adherence when doing so. Establishing the differences in service provision between types of pharmacies and why people choose a pharmacy to frequent should be a focus of future research.

## 1. Introduction

The failure of patients to adhere to their medication regimes is a multifaceted issue faced by all clinicians. The most thorough and well-thought-out treatment plans are ineffective if the patients do not take the medication as agreed upon. Medication adherence is a behaviour that can be defined as the degree to which the patient follows a mutually agreed upon course of treatment [1,2]. The literature generally accepts a person to be adherent if they take the medication as prescribed at least 80% of the time [3,4]. Medication adherence is a significant global issue: the World Health Organisation estimated that rates are as low as 50% in certain patient groups [1,3,5]. There are a variety of reasons why individuals do not adhere to their medication: forgetfulness, lack of access, cost, or experiencing side effects are some factors identified [1,5]. Poor adherence contributes to treatment failure and deteriorating health outcomes for patients [2,6].

**Figure 1:**
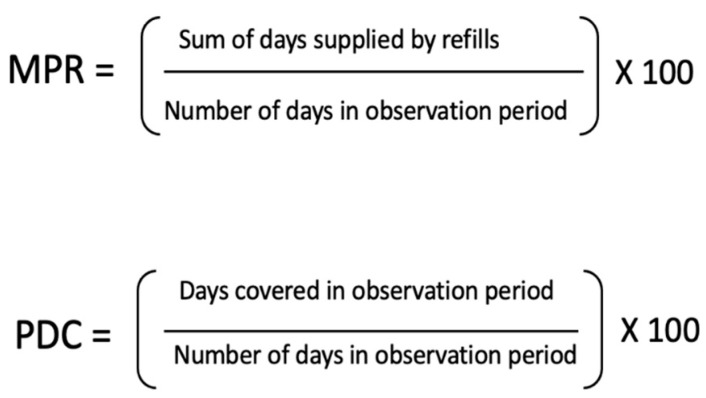
MPR and PDC calculations.

There are a range of methods used to measure medication adherence, such as measuring drug metabolites, pill counts, pharmacy dispensing records, patient reports and devices that record a pill container being opened. The most common method is to use pharmacy records in large observational cohort studies. This is because analysing dispensing or prescribing records is relatively cheap, non-invasive to participants, has less participations bias, and easy to conduct on a large-scale compared to other measures. A pharmacy records the days’ worth of a medicine dispensed over a period of time which can be used to calculate when the patient has medicine available. Medication possession ratio (MPR) and proportion of days covered (PDC) are the calculations most frequently used (Figure 1). A subtle difference is that MPR can overestimate medication adherence if individuals collect their refills early. This is then reported as the pharmacies mean PDC or as a proportion of their patients with a PDC greater than 80% [7].

Patients collecting their medication from a pharmacy is generally the final step in the care pathway. Research has identified that patient-pharmacist interactions at this point have the opportunity to enhance patients’ medication adherence [5].

This review focuses on the two most studied types of pharmacies based on their ownership structure. While there is a lack of established definitions, we recognise the following.

- Independent pharmacies follow the small owner/operator model with an individual owning up to five pharmacies.
- Chain pharmacies are typically a corporation with 6 to 200+ pharmacies operating under a nationwide banner [4,8,9].

Through their greater buying power, chain pharmacies are often able to offer lower priced medications than independent pharmacies [10]. Evidence also suggests chain pharmacies offer more services although concern is being raised about a focus on profitability impacting service quality [8]. The greater patient satisfaction reported by the users of independent pharmacies is believed to be a result of a greater focus on interpersonal interactions in these businesses [11,12]. The potential impact of patient’s pharmacy choice on their medication adherence has yet to be established.

By reviewing the literature where a variety of established pharmacy models exist, countries such as New Zealand and Australia with emerging discount pharmacy chains may be able to anticipate their impact.

### Aims

This review aims to investigate if the users of chain pharmacies have greater medication adherence than the users of independent pharmacies. We will also investigate patterns in pharmacy patronage and its impact on medication adherence among patient groups, such as elderly, those with a low income, certain conditions, or those with a high medication burden. We hypothesise that the users of chain pharmacies will have greater rates of medication adherence because of the lower cost of medications.

## 2. Materials and Methods

A systematic narrative review was conducted using the EMBASE and MEDLINE(R) databases which we deemed suitable to capture our intended literature. Authors followed the Preferred Reporting Items for Systematic Reviews and Meta-Analyses (PRISMA) statement for reporting systematic reviews and meta-analyses of studies that evaluate health-care interventions. This review was not registered.

The following search was used (“patient compliance” OR “medic* adherence” OR “medic* persistence” OR “medic* possession” OR “medic* compliance”) AND (“community pharmac*”, “dispensing channel”, “retail pharmac*”) AND (“chain”, “discount”, “mass retail*”).

The asterisks allow the search engine to include any terms which begin with that combination of letters, for example, both medicine and medication will be included in a search for medic*.

The EMBASE search window was between 1947 and the 11th of March 2022 and MEDLINE(R) was between 1946 and week 1 March 2022.

### Inclusion Criteria

To be included, the study must have compared the rates of medication adherence between the users of independent and chain pharmacies. We did not exclude any papers because of the method used to measure medication adherence due to the lack of accepted definitions for the type of pharmacies, methods for measuring medication adherence, and covariates. Studies were excluded if they carried out an intervention aimed at improving medication adherence in a retail or community pharmacy without comparing it to a different type of pharmacy. Studies comparing medication adherence between specialty and community pharmacies were excluded.

We initially looked to compare mail-order pharmacies with traditional community pharmacies. However, in 2016 Fernandez et al. carried out a review that included most of the studies we had sought for retrieval for this section. As far as we are aware nothing new has been published on the topic since 2016 [13]. 

J.N. removed duplicates by hand before screening titles and then abstracts. The remaining papers were fully appraised by J.N. and A.S. who had to agree on which paper meet the inclusion criteria. During the full article review, J.N. sought out studies referenced in the article that may meet our inclusion criteria. Two articles were identified and fully appraised but only Kalsekar et al. was included in our review [14,15]. The aims, methods, and results of included papers were then extracted by J.N. J.N. and A.S. collaborated to assess and interpret the results of each paper following the PRISMA guidelines and assess the quality and potential for bias using the Critical Appraisal Skills Programme tool (CASP) [16]. No studies were excluded from our quality assessment.

## 3. Results

**Figure 2:**
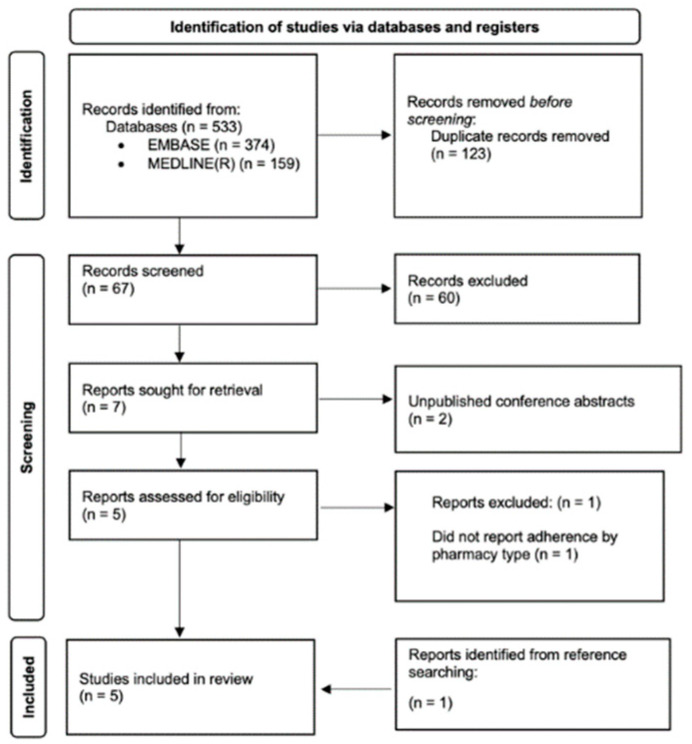
Flowchart summary of search strategy.

Our search identified 410 potential articles. A total of seven were sought for full-text review, after which four articles were deemed to be eligible. Two further studies were found from reviewing the eligible papers’ references, one of which was included in this review, as shown in the PRISMA flow chart (Figure 2). The main results are summarised below in Table 1.

### 3.1. Quality Assessment

The studies were generally assessed to be of high quality; however, several areas of weakness were identified. Firstly, three studies failed to state how the type of pharmacy was defined, thus reducing the generalisability of the results [14]. Another potential issue was the methods used to assign what type of pharmacy someone uses. By assigning someone to the type of pharmacy they collected the first prescription from, which two studies did, they do not account for people’s behaviour for the remainder of the study period [4,14]. Two separate studies reported the medication adherence for individual pharmacies but it is unclear how they accounted for participants using other pharmacies during the follow up period, they also the results were presented in a manner which made it difficult to interpret [17,18]. The completed CASP table may be viewed in the Appendix A (Table A1).

### 3.2. Summary of Findings

The majority of the included studies took place in North America, followed patients’ medication adherence for 12 months using pharmacy records data, and were all published in the past 15 years. Two of the studies reported medication adherence at a pharmacy level and one used a self-reported measure of medication adherence.

Varying rates of medication adherence were seen across the five studies. Kalsekar et al. found independent pharmacy users to have a mean MPR for oral anti diabetic medications of 0.90 (95% CI 0.89–0.91) compared to 0.88 (95% CI 0.87–0.89) for chain pharmacy users, a statistically significant difference [14]. Urick et al. also observed that independent pharmacy users had greater medication adherence to oral anti diabetic medications as well as a custom measure of chronic medications to the average pharmacy, however, there was no significant difference for statins and renin-angiotensin system antagonists (RASA) [18]. Urick et al. created the custom measure of chronic medicine by calculating the PDC for 71 medicine classes related to a range of common chronic diseases to assess patients overall medication adherence [19]. Evans et al. found the proportion of new statin patients with >80% PDC over a 12 month period was 55.0% for the users of chain pharmacies and 54.1% for the users of independent pharmacies, although the observed difference was not statistically significant [4]. Jacobs et al. were unable to detect a difference in self-reported medication adherence between different types of pharmacy [8]. In contrast, Desai et al. found the users of chain pharmacies to have the greatest medication adherence [17]. Desai et al. ranked pharmacies by mean PDC and categorised the top 50% as high performers and the bottom 50% to be low performers. The odds ratio of an independent pharmacy being a low performer compared to chain pharmacies was found to be 1.23 (95% CI 1.10–1.37), 1.68 (95% CI 1.56–1.80), and 1.47 (95% CI 1.37–1.58) for oral anti diabetic medications, RASA, and statins, respectively [17].

While the included studies had comparable objectives and methods, variance in the sample populations and secondary outcomes, as shown in Table 1, allows us to comment on the possibility that the different pharmacy types are unequally impacting certain groups of people. We have identified four themes where medication adherence is potentially being unequally affected by different types of pharmacies.

### 3.3. Low Income

Several studies concluded that low income could predict poor medication adherence [4,17]. Desai et al. reported that a pharmacy located in a county with a low median income was more likely to be categorised as a low medication adherence performer [17]. Evans et al. found that low-income drug coverage could predict low adherence with an adjusted odds ratio of 0.81 (95% CI 0.71–0.92), compared to those without known low-income drug coverage [4]. They also reported that a greater proportion of low-income patients used independent pharmacies over the generally cheaper chain pharmacies [4].

Two studies took their samples from Medicaid data, a program designed to provide health coverage to low-income Americans [14,20]. Both found independent pharmacy users to have greater medication adherence than chain pharmacy users suggesting that low-income patients have greater medication adherence when using independent pharmacies.

### 3.4. Medication Burden

Medication burden can be defined as the impact of healthcare on a patient’s function and well-being [21]. The users of independent pharmacies were found to have a greater medication burden than the users of chain pharmacies [4,14]. Kalsekar et al. reported that independent pharmacy users had significantly more prescriptions for chronic conditions dispensed than the users of chain pharmacies over a 12 month period [14]. Evans et al. found that independent pharmacy users concurrently had more classes of medications dispensed in the year preceding the observation period than the users of other types of pharmacies [4].

Evans et al. reported that patients collecting one or more medications, in addition to the statin that was studied, are more likely to be adherent to their medication regime [4]. Similarly, Kaleskar et al. found the number of prescriptions for chronic medications someone collected to be positively associated with medication adherence [14].

### 3.5. Age

Kalsekar et al. and Jacobs et al. both reported that medication adherence increases as individuals age increases [8]. Similarly, Evans et al. also found that those over 65 years old had greater medication adherence, than those under the age of 65 years, irrespective of the type of pharmacy they use, although a greater portion of them chose to use independent pharmacies over chain pharmacies [4].

Kalsekar et al. and Desai et al.’s sample selection gives some insight into the impact the type of pharmacy someone uses has on their medication adherence depending on their age [14,17]. Kalsekar et al. only included under the age of 65 years old and reported the users of independent pharmacies to have greater medication adherence than the users of chain pharmacies [14]. In contrast to Desai et al. used Medicare data which produced a sample largely composed of over 65 year olds [17]. To be eligible for Medicare you must be over the age of 65 years old, have a qualifying disability or permanent kidney failure [22]. Desai et al. reported significant odds ratios of independent pharmacy users having poor medication adherence compared to the users of chain pharmacies [17].

### 3.6. Medications

Oral anti diabetic medications were investigated by several papers [14,17,18]. Urick et al. found patients to have greater medication adherence to this class of medications when using independent pharmacies. Kaleskar et al. only investigated oral anti diabetic medications and found adherence rates to be greater for users of independent pharmacies. Desai et al.’s findings were in contrast, reporting an odds ratio of 1.23 (95% CI 1.10–1.37) that an independent pharmacy would be a low performer for adherence to oral anti diabetic medications.

Statin adherence was reported by 3 of our studies [4,17,18]. Desai et al. found independent pharmacy patrons to have worse statin adherence. The odds ratio of an independent pharmacy being a low performer for statin adherence was 1.47 (95% CI 1.37–1.58) compared to a chain pharmacy. However, Urick et al. and Evans et al.’s did not find a significant difference in statin adherence between the users of independent and chain pharmacies.

However, Urick et al. disagreed with Desai et al. regarding RASA adherence. Urick et al. found that using an independent pharmacy did not have a significant effect on RASA adherence. In contrast, Desai et al. showed chain pharmacy users to have great RASA adherence, reporting the odds ratio of an independent pharmacy being a poor performer to be 1.68 (95% CI 1.56–1.80) compared to a chain pharmacy.

## 4. Discussion

The five studies included in this review could not conclude that chain pharmacy users had greater rates of adherence than the users of independent pharmacies. However, those with a low income, high medication burden, increased age, or taking specific medications appear to be disproportionately impacted by the type of pharmacy they use. Exploring these themes may expand on how the type of pharmacy someone uses affects their medication adherence.

### 4.1. Low-Income

Those with a low-income tend to use independent pharmacies more frequently than chain pharmacies and potentially have better medication adherence when they do so. This is demonstrated by the two studies whose samples were largely composed of beneficiaries reporting that independent pharmacy users have greater medication adherence [14,18].

The cost of medications can be a major barrier to medication adherence and is a key difference between chain and independent pharmacies [5]. A 2015 cross-sectional study of 60,000 pharmacies across the United States by Luo et al. found the cost of medications in independent pharmacies to be 1.61 (95% CI 1.58–1.64) times that of a large pharmacy chain [10]. We postulated that those with a lower income would have a greater demand for low-cost medications leading them to frequent chain pharmacies. However, that is not what the findings of this review have observed.

The two studies, Evans et al. and Desai et al., which investigated the effects of income agreed with the literature that low income is a predictor of low medication adherence [3,5,23,24,25].

Education is a variable potentially linking income and medication adherence. A lower level of education is associated with both a lower income and lower health literacy [26,27]. Health literacy describes a person’s ability to interpret health information and use it to make decisions about their care [28]. Poor health literacy has been shown to lead to lower medication adherence [5,25,29]. Someone with poor health literacy may experience greater improvements in their medication adherence from high quality interactions with a pharmacist, as the literature suggests occurs in independent pharmacies, than receiving cheaper medication. Hence, our studies report that low-income patients have better medication adherence in independent pharmacies [30,31].

### 4.2. Medication Burden

Two studies reported that independent pharmacy users had a greater medication burden than the users of chain pharmacies [4,14].

The findings of this review contrast with the literature, as our included studies reported greater medication adherence among individuals with a higher number of medications [3,5].

These findings were unexpected as patients with a high medication burden may be more likely to have medication changes to improve their therapy. This effect may compound if the patients are non-adherent, as the prescriber may be more likely to augment or adjust the patient’s medications furthering their medication burden and the complexity of their regime which in turn makes it harder to adhere to the medication regime. In addition, a known weakness of measuring medication adherence using pharmacy record data is that an individual starting and stopping medications throughout the follow up period will cause the patient to be recorded as non-adherent regardless of whether the prescriber leads the change or not.

By having a higher proportion of patients with a high medication burden independent pharmacies’ are facing more complex patient care demands [5].

### 4.3. Age

Evans et al. reported that older individuals tend to use independent pharmacies, which is supported by the literature [32,33]. However, three of our studies agreed that older individuals have greater medication adherence which is met with some conjecture from the literature [4,8,14]. A 2013 systematic review of 51 studies into medication adherence reported age as having an inconsistent impact on medication adherence [1,5,29]. The potential for different types of pharmacies to affect adherence unevenly depending on the patient’s age is worthy of future study.

### 4.4. Medications

There are several medication specific differences observed in the studies we have included. Oral anti diabetic medications tended to have greater adherence at independent pharmacies while RASA and statins tended to have better adherence at chain pharmacies [4,14,17,18].

When treating uncontrolled diabetes best practice recommends augmenting therapy with another oral anti diabetic medication class creating a more complex regime [34]. In contrast, RASA or statin therapy is less complex and would typically only involve one medication from each class [35,36]. Similar to the medication burden section, patients with more complex medication regimes may be benefiting from the additional pharmacist service observed in independent pharmacies [5]. 

Independent pharmacy users tend to have a lower income, be older, and take more medications than the users of chain pharmacies. These themes suggest the users of independent pharmacies are biased face more barriers to medication adherence. However, that is not what is being observed which postulates that independent pharmacies are positively influencing their user’s adherence.

### 4.5. Limitations

The themes discussed in this review is not an exhaustive list, as many other factors affect a person’s adherence [5]. Further research into who uses different types of pharmacies and how individuals decide which pharmacies to frequent would advance our understanding of this issue.

The lack of information describing how differ pharmacies may reduce the generalisability of this review since we do not know how the studied pharmacies compare to the those, we wish to compare them to.

Most medication adherence studies focus on chronic conditions because they generally require lifelong treatment, have serious consequences for non-adherence, and are the easiest to measure medication adherence on a large scale [25]. There is a lack of research investigating the impact pharmacy type has on adherence to short term medication.

All of the included studies except Jacobs et al. used pharmacy records data to calculate their measures of medication adherence. An established limitation of this methodology is that it is assumed that a medication dispensed is a medication taken [37]. However, it is possible that the portion of medication dispensed but not taken differs between independent and chain pharmacy users. Paying less for a medication may reduce the perceived value of the medication making it less likely that they take it after having it dispensed, we believe this is an area worthy of further research.

Like any review, these findings may be impacted by bias surrounding the decision to publish research, particularly due to the business implications of this area of study.

## 5. Conclusions

The findings of this review were unable to confirm our hypothesis that the users of chain pharmacies have greater medication adherence than those who used independent pharmacies. Patient characteristics such as low income, high medication burden, and age appear to be associated with both poor medication adherence and frequenting independent pharmacies more than chain pharmacies. However, independent pharmacy users’ medication adherence is at least comparable to those who use chain pharmacies. There may be an unknown factor that draws adherent patients to independent pharmacies or a difference in the service independent pharmacies provide which improves medication adherence. Establishing the differences between independent and chain pharmacies and investigating how different groups’ preferences drive them towards different types of pharmacies could help improve patients’ medication adherence and health outcomes.

## Figures and Tables

**Table 1:** Summary of included studies.

Author	Data Source	Medications Investigated	Sample Size	Pharmacy Assignment	Observational Period	Patients’ Inclusion Criteria	Adherence Measure	Numerical Results
Kalsekar et al., 2007 [14]	Medicaid Indianapolis, US.	Oral anti diabetics	2696 patients	Pharmacy first script collected from	1 year	New medication, aged < 65	Medication possession ratio (MPR)	Mean MPR: independent 90% and chain 88%
Evans et al., 2009 [4]	Drug Plan and Extended Benefits Saskatchewan, Canada.	Statins	8699 patients	Pharmacy first script collected from	1 to 3 years	New medication	Proportion of patients with a proportion of days covered (PDC) > 80%	Proportion of patients with a PDC > 80%: Independent 54.1%, mass merchandise 47.8%, and chain 55.0%
Desai et al., 2016 [17]	Medicare County-level throughout the United States	Oral anti diabetics, renin-angiotensin system antagonists (RASA’s), and statins	28,969 pharmacies (117 million patients)	Type of pharmacy frequented	6 months	NA	PDC used to rank pharmacies performance as either a high performer (top 50%) or low performer (bottom 50%) by drug class	Odds ratio of the users of an independent pharmacy being a “low performer” anti diabetics 1.23, RASA’s 1.68, and statins 1.47
Urick et al., 2020 [18]	Medicaid North Carolina, United States.	Statins, RASA’s, oral anti diabetics, and a chronic medication measure	2139 pharmacies	Pharmacy from which at least 80% of chronic medications were collected during a 3 month period	12 month rolling period starting from the first dispensing	Patient needed at least 2 fills for the given medication	Proportion of patients with a PDC > 80%	Pharmacies which are independent have better adherence to chronic medications and oral anti diabetics than an average pharmacy. No significant difference for RASA and statins.
Jacobs et al., 2020 [8]	Pharmacies from 9 geographical areas around the United Kingdom	All medications	775 patients	Pharmacy attended at the time of survey	NA	Survey given to 30 consecutive patients at each pharmacy, aged between 18 and 93	Score >120 on the MARS adherence questionnaire	No significant differences

## Data Availability

Not applicable.

## Appendix A

**Table A1:** CASP quality assessment.

	Are the Results of the Review Valid?						What are the Results?			Will the Results Help Locally?	
Author	Clearly Focused Issue	Cohort Recruitment	Exposure Accurately Measured to Minimise Bias	Outcome Accurately Measured to Minimise Bias	Identified and Accounted for Confounding Factors	Follow Up Was Complete and Sufficiently Long	Overall Results	Precision of Results	The Results Are Believable	Applicable to Local Population	Results Fit with Other Evidence
Kalsekar et al., 2007 [14]	Yes	Yes	Used index script to define user. Did not define pharmacy types	MPR	Yes	12 months	Yes	Statistically significant	Yes	Yes	Yes
Evans et al., 2009 [4]	Yes	Yes	Used index script to define user. Defined pharmacy types	PDC >80%	Yes	3 years	Yes	Statistically significant	Yes	Yes	Yes
Desai et al., 2016 [17]	Moderate	Yes	Type of pharmacy. Did not define pharmacies	Mean PDC of users used to rank pharmacies	Yes	6 months	Unusual measure used	Statistically significant	Yes	Moderately generalisable	Yes
Urick et al., 2020 [18]	Yes (Pharmacy type a secondary outcome)	Yes	Did not define pharmacy type	Proportion of patients with a PDC >80%	Yes	12 months	Hard to interpret	Some medications significant some not	Yes	Yes	Yes
Jacobs et al., 2020 [8]	Yes	Yes, but missed a pharmacy type which declined to participate	Yes, and clearly defined pharmacy types	Yes, self-reported adherence MARS	Yes	NA	Yes	Not statistically significant	Yes	Yes	Yes
